# A contemporary decennial examination of changing agricultural field sizes using Landsat time series data

**DOI:** 10.1002/geo2.4

**Published:** 2015-04-07

**Authors:** Emma V. White, David P. Roy

**Affiliations:** ^1^ Geospatial Sciences Center of Excellence, Wecota HallSouth Dakota State UniversityBrookingsSD 57007USA

**Keywords:** field size, agriculture, land cover land use, change, drivers

## Abstract

Field size distributions and their changes have not been studied over large areas as field size change datasets are not available. This study quantifies agricultural field size changes in a consistent manner using Landsat satellite data that also provide geographic context for the observed decadal scale changes. Growing season cloud‐free Landsat 30 m resolution images acquired from 9 to 25 years apart were used to extract field object classifications at seven sites located by examination of a global agricultural yield map, agricultural production statistics, literature review, and analysis of the imagery in the US Landsat archive. High spatial resolution data were used to illustrate issues identifying small fields that are not reliably discernible at 30 m Landsat resolution. The predominant driver of field size change was attributed by literature review. Significant field size changes were driven by different factors, including technological advancements (Argentina and USA), government land use and agricultural policies (Malaysia, Brazil, France), and political changes (Albania and Zimbabwe). While observed local field size changes were complex, the reported results suggest that median field sizes are increasing due to technological advancements and changes to government policy, but may decrease where abrupt political changes affect the agricultural sector and where pastures are converted to arable land uses. In the limited sample considered, median field sizes increased from 45% (France) to 159% (Argentina) and decreased from 47% (Brazil) to 86% (Albania). These changes imply significant impacts on landscape spatial configuration and land use diversity with ecological and biogeochemical consequences.

## Introduction

Agriculture is associated with some of the most significant human‐induced land cover land use changes, with dramatic cropland expansion in the last several hundred years and a marked increase in productivity in the past few decades driven by increasing populations and changing diets (Goldewijk and Ramankutty 2004; Kastner *et al*. 2012; Tilman *et al*. 2002). Globalisation has shortened the connections between consumers and agricultural commodities with contemporary production patterns influenced by demands from distant urban areas and by food, fuel and fibre preferences among nations (Garrett *et al*. 2013; Seto *et al*. 2012). Although demands remain high, agricultural productivity as well as cropland area is unevenly distributed globally (Foley *et al*. 2011; Lambin *et al*. 2013; Monfreda *et al*. 2008; van Asselen and Verburg 2012).

Field sizes are indicative of the degree of agricultural capital investment, mechanisation, and labour intensity (Herzog *et al*. 2006; Kuemmerle *et al*. 2013; Rodríguez and Wiegand 2009). Information on the size of fields is needed to plan and understand these factors, and may help the allocation of agricultural resources such as water, fertiliser, herbicide, and farming equipment (Anderson *et al*. 2012; Johnson 2013; Rudel *et al*. 2009; You *et al*. 2009). Field sizes are thought to be increasing due to agricultural intensification as farmers seek to maximise profit and reduce risk through larger agricultural enterprises, with ecological and biogeochemical consequences. Various national and international agencies report crop yields, and sometimes farm size statistics, but statistics concerning the sizes of agricultural fields or their changes are not reported. Recently, a *c*. 2005 global field size dataset was developed by spatial interpolation of 13 963 crowd sourced (internet and mobile phone based) geotagged categorisations of GoogleEarth images into very small, small, medium and large field size categories (Fritz *et al*. 2015). The interpolated global field size dataset has unknown accuracy and does not capture field size change. Studies of the incidence, drivers, modifiers, and impacts of changing field sizes have not been undertaken over large areas and certainly not from a global perspective.

Satellite data provide a synoptic view and have been used for agricultural applications, including cropland distribution mapping, crop condition monitoring, crop production assessment, and yield prediction (Bauer *et al*. 1978; Becker‐Reshef *et al*. 2010; Johnson and Mueller 2010). The ability of satellite data to monitor agriculture reliably is dependent on many factors but is fundamentally constrained by the satellite spatial resolution relative to the field spatial dimensions. Commercial high spatial resolution (<10 m) satellite data have only been available since 1999 (Belward and Skøien 2014; Johansen *et al*. 2008; Turker and Ozdarici 2011) and so cannot be used for field size change analysis prior to 1999. The Landsat series of satellites provides the longest satellite data record spanning from 1972 to present day (Roy *et al*. 2014) and with appropriate resolution for monitoring anthropogenic surface changes (Hansen and Loveland 2012; Townshend and Justice 1988). The recent free availability of the Landsat data in the US Landsat archive (Wulder *et al*. 2012) provides the opportunity to study field size changes for large areas and in a globally distributed and consistent way. Landsat‐based agricultural applications were developed after the launch of the first Landsat in 1972 and have been subject to multi‐agency funded support through initiatives such as the Large Area Crop Inventory Experiment (LACIE) (MacDonald *et al*. 1975) and US Department of Agriculture (USDA) initiatives (Hanuschak *et al*. 1980; Johnson and Mueller 2010). A seminal agricultural field size study was undertaken by digitising more than 112 000 US and Canadian agricultural field boundaries from Landsat data sensed from 1977 through 1980 (Ferguson *et al*. 1986).

This study, for the first time, quantifies agricultural field size changes. Satellite data are used to extract field sizes and to provide geographic context for the spatial nature of observed changes and literature review is used to attribute the change drivers. A pragmatic approach is used to select Landsat images at locations where agricultural field size changes are discernible. Major global agricultural production regions are identified by analysis of 2010 FAO continental crop production statistics (FAOSTAT 2010) and global 5 min EarthStat crop yield data (Monfreda *et al*. 2008). Within these regions, pairs of cloud‐free growing season Landsat images acquired about a decade apart at locations with documented changes in field size, farm size, agricultural intensity, or extent are selected. GoogleEarth high spatial resolution data are used to help ensure that only crop fields are examined. In addition, Landsat image pairs are selected where rapid political changes caused significant agricultural sector change. Fourteen Landsat images sensed up to 25 years apart at seven locations that each include a major cereal or biofuel crop are considered. High spatial resolution 2.5 m Quickbird 2 data are used to illustrate difficulties in identifying small field sizes that are not discernible at 30 m Landsat resolution. The implications of observed field size changes on landscape spatial configuration and land use diversity and their ecological and biogeochemical consequences are considered briefly, and recommendations for future research discussed.

## Materials and methods

### Satellite data

**Figure 1:**
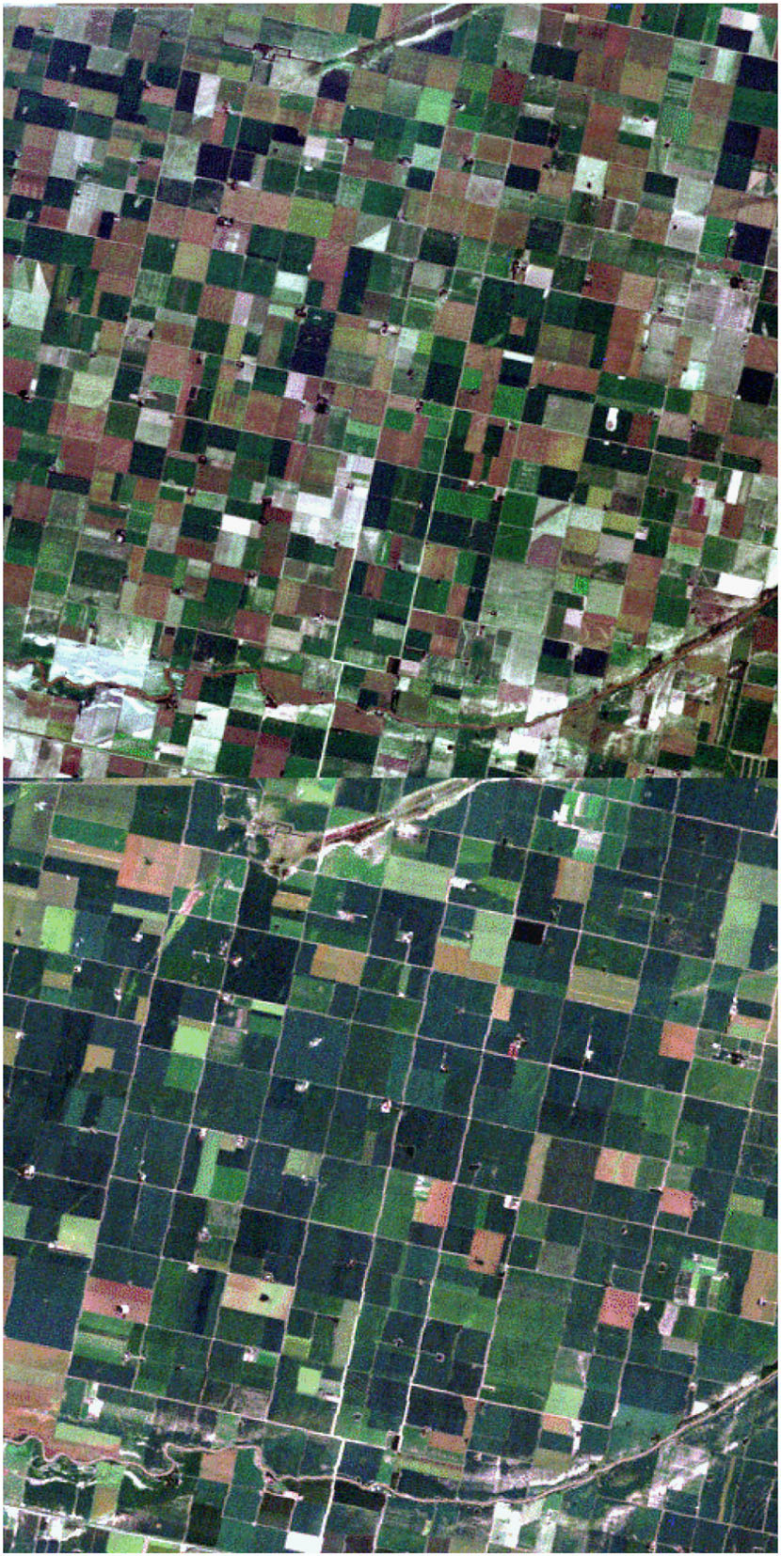
True colour (red, green, blue wavelength) 30 m top of atmosphere Landsat 5 TM reflectance images for a 15 × 15 km (500 by 500 30 m pixel) area near Los Surgentes, Córdoba, Argentina. Landsat path 228, row 83, acquired 10 February 1986 (top) and 15 February 2011 (bottom)

Growing season cloud‐free Landsat 30 m resolution images acquired from 9 to 25 years apart were used to examine field size changes. Global 30 m multispectral Landsat observations have been provided by the Landsat 4 and 5 Thematic Mapper (TM), Landsat 7 Enhanced Thematic Mapper Plus (ETM+), and Landsat 8 Operational Landsat Imager (OLI) from 1984 to present (Loveland and Dwyer 2012; Roy *et al*. 2014). These sensors provide multispectral observations in similar reflective wavelength bands, in the visible, near‐infrared and middle‐infrared that can be used to identify landscape features such as fields and their boundaries (Yan and Roy 2014). For example, Figure 1 illustrates qualitatively the utility of 30 m Landsat data to monitor field size change over a 25‐year period.

The Landsat data were obtained from the United States Geological Survey (USGS) Earth Resources Observation and Science (EROS) via the Global Visualisation Viewer internet system (GloVis: http://glovis.usgs.gov/). Selected Landsat data were converted from digital numbers to top of atmosphere reflectance and brightness temperature using Landsat acquisition specific calibration information and standard processing algorithms to ensure temporally consistent data needed for their multi‐temporal comparison (Roy *et al*. 2010). These data are made available in approximately 180 × 170 km scenes defined in a Worldwide Reference System of path (groundtrack parallel) and row (latitude parallel) coordinates (Arvidson *et al*. 2001). The Landsat sensor geometry and orbit characteristics are such that each path/row can be sensed every 16 days, however globally not every scene is stored in the US Landsat archive due to a number of factors and because of cloud obscuration at the time of satellite overpass (Kovalskyy and Roy 2013). The Landsat 5 TM was launched in 1984 and remained operational until 2011. The Landsat 7 ETM+ was launched in 1999 and remains operational with an overpass 8 days later than Landsat 5 TM. In May 2003 the Landsat 7 ETM+ scan line corrector failed, systematically reducing the amount of useable ETM+ image data by 22% (Markham *et al*. 2004) and so limiting the area over which reliable mapping can be performed. In this study 30 m Landsat 5 TM from 1984 to present and Landsat ETM+ data from 1999 to 2002, i.e. before the ETM+ scan line corrector failure, were examined.

High spatial resolution data were used to illustrate the difficulties in identifying small field sizes that are not discernible at Landsat resolution. High spatial resolution data are well suited for interpretation of agricultural fields, in particular small and irregularly shaped fields, but are not available before 1999 (Johansen *et al*. 2008; Turker and Ozdarici 2011). In this study therefore a small number of Quickbird‐2 high spatial resolution 2.5 m red, green, blue and near‐infrared wavelength images (Johansen *et al*. 2008) were used to illustrate Landsat field size detection issues.

GoogleEarth time series imagery (http://www.google.com/earth/) are freely available and have near global coverage. They were used in this study as they include high spatial resolution satellite and airborne images from a variety of commercial providers and US government agencies. Only image pictures are available and the sensor characteristics are undefined so, although useful for visualisation and contextual interpretation (Hansen *et al*. 2014), the imagery cannot be reliably processed using standard remote sensing algorithms. In this study GoogleEarth time series imagery were used to help visually confirm that fields identified in the Landsat data were arable and not pasture or other grassland uses which can be hard to discriminate using just Landsat data (Müller *et al*. 2015). In addition, GoogleEarth imagery was used to examine rice fields.

### Agricultural statistics

Agricultural statistics extracted from the 2010 global FAO crop commodity production statistical database (FAOSTAT 2010) were used to identify the top four harvested staple cereal crops, namely, wheat, maize, rice, and soybeans, that together account for more than 50% of the total global harvested acreage and production (FAOSTAT 2010). In addition statistics for the top producing non‐cereal biofuel crops, i.e. oil palm and sugar cane, were extracted as these crops have experienced rapid development in the last several decades (Cassidy *et al*. 2013; Goldemberg 2008; Graham‐Rowe 2011). The FAO crop data were collated using the best available information provided by national governments, online databases, publications and questionnaires, and unofficial sources and provide the most complete publically available resource for global agricultural crop production (FAOSTAT 2010). The FAO data were used to identify the global rank by crop production for each of the six crops at continental scale.

For the USA, county level statistics produced by the USDA agricultural census were also examined. The census is undertaken every 5 years by classification of aerial survey photography, field sampling and by mailing a questionnaire to a sample of farmers with holdings that produce and sell at least $1000 of agricultural products per year (Johnson 2013; USDA 2009). County level mean farm size statistics are reported but the farm area definition includes the area of land used for livestock agriculture and on‐farm infrastructure. Therefore, in order to derive a US metric more compatible with this research, an alternate measure of mean farm size per county was calculated by dividing the total harvested cropland area per county by the number of farms with harvested cropland per county. Percent county level mean farm size change was computed using the 1987 and 2007 census data.

### Global crop yield map

**Figure 2:**
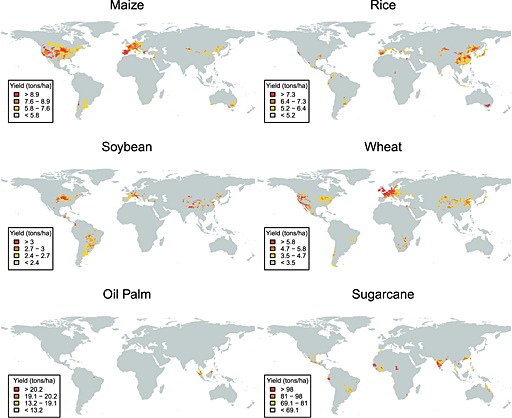
Global high‐yield agricultural regions for staple cereal crops (maize, rice, wheat, and soybeans) and biofuel crops (oil palm and sugarcane). The 5 min EarthStat crop yield data were used to derive the illustrated global crop yield percentiles coloured as red (>95th percentile), orange (>90th to 95th percentile), yellow (>80th to 90th percentile), grey (≤80th percentile or undefined)

The global 5 min EarthStat crop yield data (Monfreda *et al*. 2008) were used to map the major global agricultural yield regions for the four harvested staple cereal and the two biofuel crops. The EarthStat data are derived by spatial disaggregation of national and sub‐national scale 1990–2003 agricultural census information into cultivated cropland areas defined by Ramankutty *et al*. (2008). The data, acquired from EarthStat (http://www.earthstat.org/) include a quality layer that designates if the data were derived at (1) county level, (2) state level, (3) interpolated from the nearest county or state level data within 2 degrees, (4) national level, or (5) were not defined. In this study only sub‐national scale data [i.e. (1) to (3)] were used as the national scale data do not provide sufficiently detailed spatial information. Figure 2 shows the 80%, 90%, and 95% crop yield percentiles for the four cereal and two biofuel crops.

### Location of Landsat scenes capturing contemporary agricultural field size change

The globe was divided into five continental regions (Africa, Americas, Asia, Europe and Oceania) and the 2010 global FAO statistics were used to identify the top two continents by production (tons) for each of the six crop types. Antarctica was not considered as it has no cropland. Within the chosen continents, only regions where the global crop yield exceeded the 80th percentile (coloured, Figure 2) were considered. Selection was further refined within these major production regions by review of the post 1980 published agricultural literature concerning documented changes in field size, farm size, and agricultural intensity or extent. Priority for selection was given to agricultural regions where the literature described information on field and farm size change. If the literature on these aspects were limited then regions were selected where there were documented changes in agricultural extent and then agricultural intensity. For example, in the USA, the percentage county level mean farm size change was used to identify counties where farm size (and so potentially field size) changes were maximal. Additional regions that were reported in the literature to have significant changes in field size or agricultural productivity that were not evident in the global crop yield map or in the FAO statistics were included. These were in nations that experienced rapid political changes that dramatically altered the agricultural sector.

The literature review helped attribute primary field size change drivers that were broadly categorised as being associated with technological advancements, with government land use and agricultural policy changes, and with political changes (Brown and Schulte 2011; Hazell and Wood 2008; Muller and Munroe 2008). When several regions could be selected with the same crop type an attempt was made to select those that were representative of different primary driving forces. A more sophisticated categorisation was not undertaken given the relatively small number of Landsat locations that could be considered and because of the complexity of understanding land cover land use changes (Turner *et al*. 2007; Veldkamp and Lambin 2001).

A single Landsat 180 × 170 km path/row was chosen within each selected primary crop production region. There were typically several suitable Landsat path/row locations in each selected region. Only path/row locations where approximately decadal growing season image pairs acquired with approximately the same calendar date were considered. This was required as field boundaries may not be spectrally separable from field interiors at different phenological stages (Ozdogan and Woodcock 2006; Pan *et al*. 2012; Rydberg and Borgefors 2001; Yan and Roy 2014). The image selection was also constrained by limited Landsat data availability in the US Landsat archive, especially for geographic regions outside of the USA, and because of cloud obscuration (Kovalskyy and Roy 2013). Only Landsat path/row locations with suitable images available in the growing season and with cloud cover less than 30%, as defined by the Landsat metadata and indicated in the GloVis Landsat ordering system, were considered. GoogleEarth high spatial resolution data were used to check that the Landsat data contained crop fields and not grasslands, although this was not always possible where there were no high spatial resolution data in GoogleEarth and was not possible for the pre‐1999 Landsat scenes.

### Landsat subset selection

From each selected Landsat image pair a 15 × 15 km spatial subset that was representative of the majority agricultural land use in the image and where obvious field size change was most visually evident was selected. This was a subjective and time‐consuming process. The subset size was kept small due to the time‐consuming nature of the field extraction process described below. The 15 km subset side dimension was selected as it is greater than the largest field dimensions reported in the literature that report long‐axis field dimensions as great as 6 km in the USA (Connor *et al*. 2011; Ferguson *et al*. 1986). The 15 km dimension was more than an order of magnitude greater than the largest field size dimensions observed in the Landsat data selected in this study.

### Landsat field extraction

Field boundaries can often be identified by visual inspection of appropriately displayed Landsat data (e.g. Figure 1) and can be straightforward to extract, especially if undertaken by a capable interpreter, for example, by screen digitising or by interactive thresholding spectral band indices (Basnyat *et al*. 2004; Lobell *et al*. 2003). Ideally, seasonal Landsat data acquired in the same year would be used to better capture seasonal agricultural differences (Lo *et al*. 1986; Schriever and Congalton 1993), and enable more clear differentiation between, for example, cropland and managed grasslands (Kuemmerle *et al*. 2006; Prishchepov *et al*. 2012), and provide improved field boundary delineation (Yan and Roy 2014). However, the availability of decadal pairs of seasonal Landsat data over suitable field size change locations was limited, particularly for Asia and Africa. Consequently, in this study, fields were extracted independently from single date Landsat images acquired about a decade apart. An interactive object based classification approach that is directly applicable to the extraction of discrete objects such as agricultural fields (Blaschke *et al*. 2014) was used. Only the field boundaries, and not the field crop types, were extracted.

Each 15 × 15 km Landsat subset was segmented using the Definiens eCognition multispectral image segmentation package (Definiens 2009). Different surface features within a subset can be identified by their remotely sensed spectral signatures (the amount of electromagnetic radiation that they reflect at different wavelengths). Due to different phenological stages certain field boundaries, such as grass strips and irrigation ditches, were not always clearly spectrally separable from the field interiors (Ozdogan and Woodcock 2006; Yan and Roy 2014). Moreover, within‐field spectral variability, caused by spatial variations in factors such as soil moisture, salinity, fertility and nutrient limitations, pesticide, herbicide and fertiliser treatments, pollution, pests and diseases, reduce field boundary separability (Chang *et al*. 2007; Hall and Badhwar 1987; Rao 2008). Consequently, the Landsat subsets were purposefully over segmented so that there were many segments per field that were then subsequently merged together into single field objects. This approach reflects standard practice; most object‐based classifiers over‐segment the scene prior to merging (Pavlidis and Liow 1990; Rydberg and Borgefors 2001). The eCognition software was used to group adjacent pixels with similar spectral signatures together, considering the six available reflective wavelength Landsat bands and by setting object shape and compactness segmentation parameters. The shape and compactness parameters were different for each Landsat subset as field sizes and shapes varied considerably (parameters ranged between 0.3 and 0.9 for shape and between 0.3 and 0.5 for compactness). The segments were merged into distinct unambiguous objects by interactive on‐screen selection. The objects were then classified as agricultural fields or other objects using the nearest neighbour eCognition supervised classifier. The classifier requires training data which were generated by selecting a representative sample of objects that were unambiguously visually identified as agricultural or non‐agricultural. The classifier was applied to the Landsat spectral band values averaged over each object to classify them into agricultural fields and other objects of no interest to this study. This process was iterated and more training samples were added as needed to provide a visually unambiguous classification. Agricultural field objects that did not fall entirely within the 15 × 15 km Landsat subset were removed.

**Figure 3:**
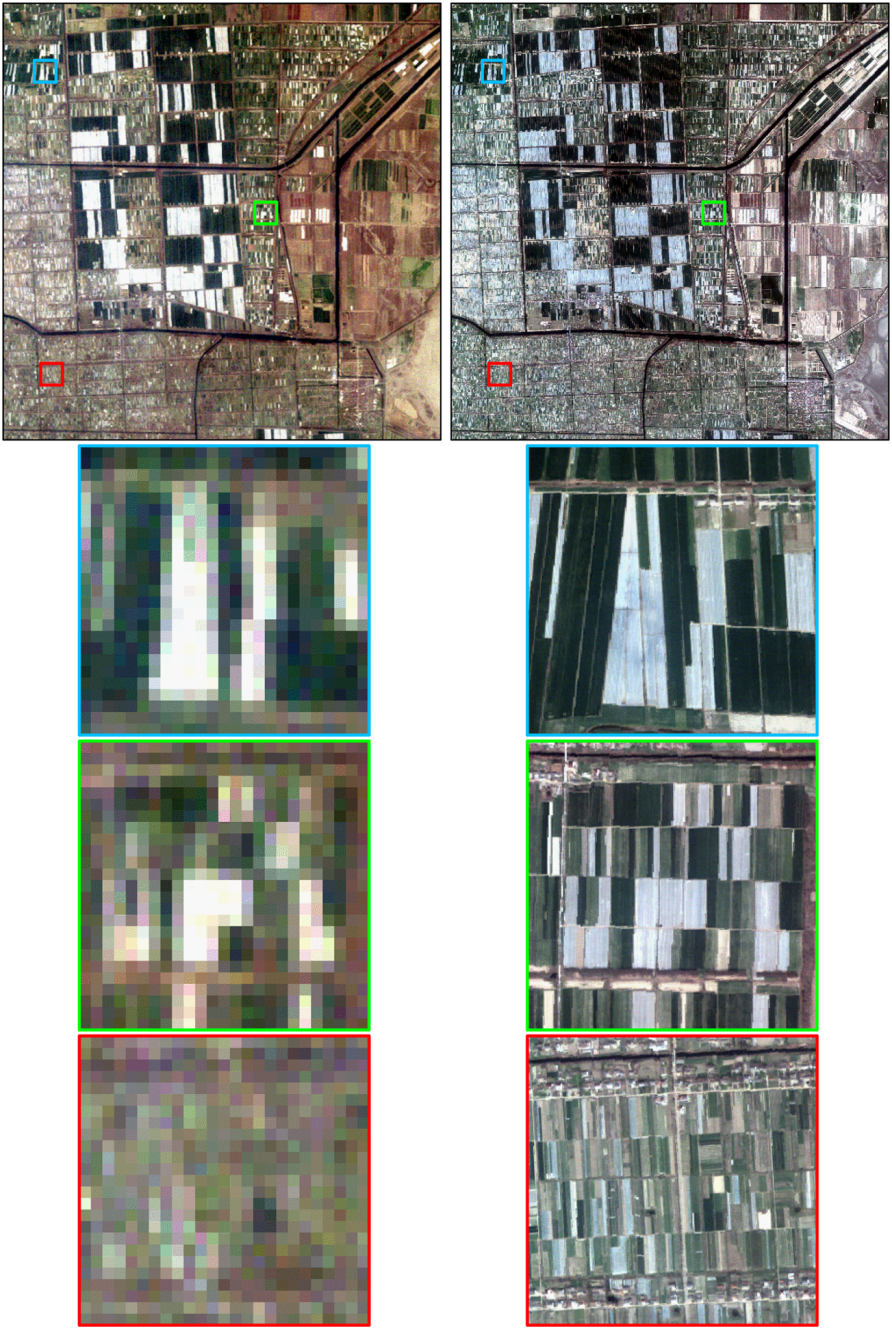
Comparison of Landsat 5 TM 30 m (left column) and Quickbird‐2 2.5 m (right column) true colour satellite data over rice paddies in Jiangsu province, Southeastern China (latitude 32.79°, longitude 120.77°). Data were acquired on 23 March 2005 (Landsat) and 7 April 2005 (Quickbird). The top row shows the same 15 × 15 km area and the boundaries of three 750 × 750 m detailed subsets, and the bottom three rows show the subsets in detail

**Figure 4:**
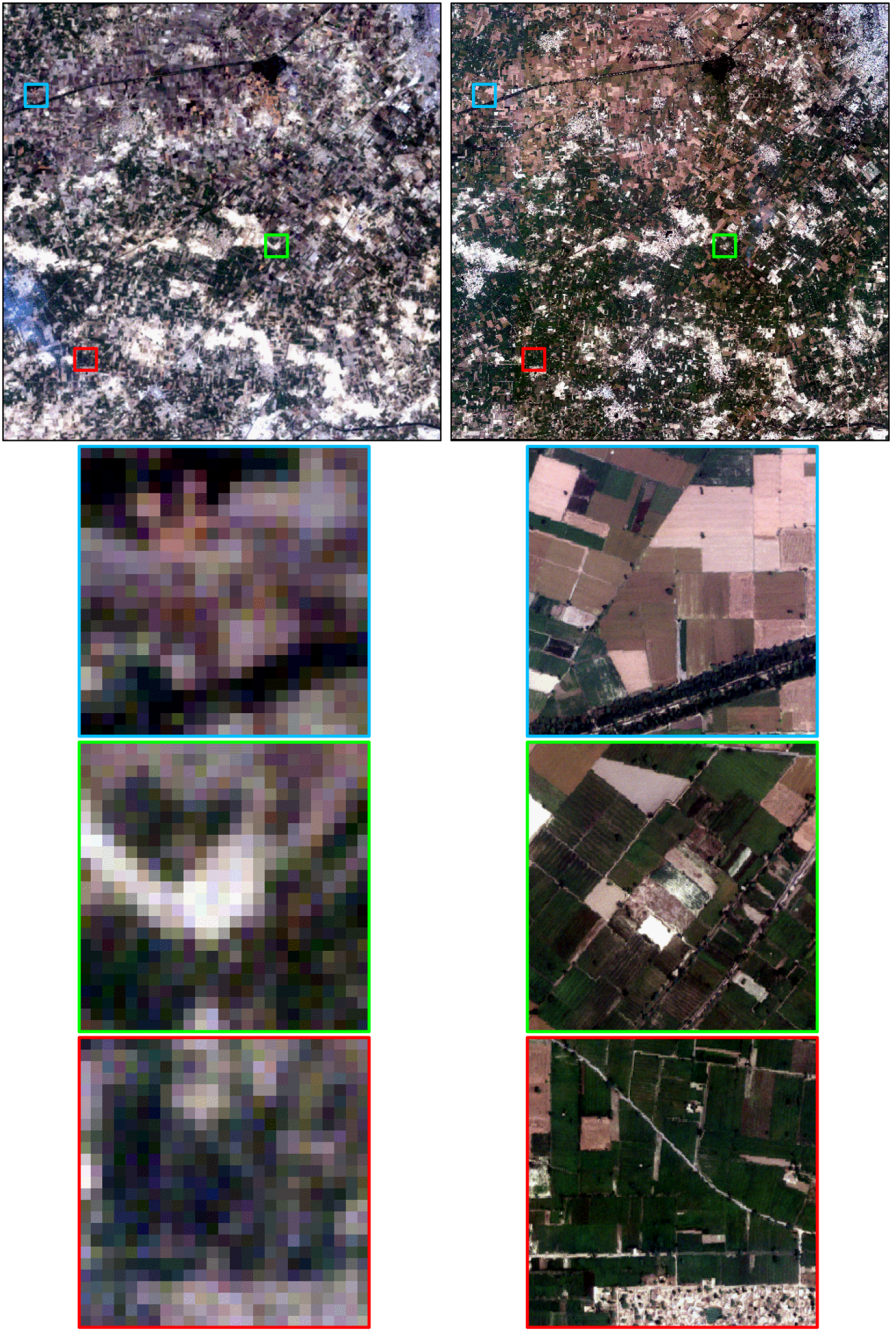
Comparison of Landsat 7 ETM+ 30 m (left column) and Quickbird‐2 2.5 m (right column) true colour satellite data over rice and wheat in north western India in the Punjab (latitude 30.14°, longitude 74.86°). Data were acquired on 28 October 2002 (Landsat) and 7 October 2003 (Quickbird). The top row shows the same 15 × 15 km area and the boundaries of three 750 × 750 m detailed subsets, and the bottom three rows show the subsets in detail

Field objects that were too small to be mapped reliably were removed. The minimum field size that can be reliably extracted from satellite data is dependent on factors including the sensor spatial resolution, satellite geolocation errors, the spectral contrast between field interiors and exteriors, and the field shape (Duveiller and Defourny 2010; Ji 1996; Mueller *et al*. 2004; Ozdogan and Woodcock 2006; Rydberg and Borgefors 2001; Yan and Roy 2014). Figures 3 and 4 show example 30 m Landsat (left columns) and 2.5 m Quickbird (right columns) images that illustrate fields that are not clearly discernible in Landsat imagery. The top rows of these figures show 15 × 15 km images and the rows below show illustrative 750 × 750 m details. The China images (Figure 3) were sensed over rice paddies in Jiangsu province, southeastern China, where irrigation systems and fertiliser inputs contribute to high rice yields (Jing *et al.* 2007) with a typical paddy rice and then winter wheat or rapeseed rotation (Xiao *et al.* 2006). The India images (Figure 4) were sensed over north‐western India in the Punjab where traditional small‐scale intensive farming has kept field sizes small despite the adoption of new irrigation technologies and new improved varieties of rice and wheat (Sampath 1992; Smale *et al*. 2008). The China Landsat and Quickbird data were acquired only one month apart and show unambiguously that fields with small axis dimensions less than two 30 m Landsat pixels cannot be discerned in the Landsat data. The China Quickbird 750 × 750 m detail images illustrate typical rice paddies that are narrow in one axis and long in the other and include fields with dimensions as little as about 10 m and 60 m in the small and long axis dimensions, respectively. The India Landsat and Quickbird data were acquired in the same month but one year apart due to limited satellite data availability, and exhibit inter‐annual variation in crop planting and harvesting. The India Quickbird data include distinct fields that are often more than two Landsat pixels wide but are sometimes not separable in the Landsat data, particularly where adjacent fields are similar and not separated by large and/or distinct boundaries. These issues were observed by Yan and Roy (2014) who adopted a conservative minimum field size extraction of sixteen 30 m Landsat pixels for their automated US Landsat field extraction algorithm research. As the field extraction methodology used in this study is interactive and includes visual assessment, a smaller minimum field size was used. Based on the results illustrated in Figures 3 and 4, and upon our experience examining the field extraction results applied to the selected Landsat data, a minimum field size mapping unit of six 30 m pixels was used. Thus, Landsat extracted field objects composed of less than six 30 m pixels were removed, i.e. the smallest extracted field size corresponded to 0.0054 km^2^.

The absolute accuracy of the resulting field extractions was unknown but, given the interactive and visual extraction approach, the field segmentations reflect the highest accuracy we judged possible. Conventionally the accuracy of satellite products is assessed by comparison with independent reference data (Justice *et al*. 2000). However, the earlier Landsat images were acquired before the availability of independent reference data, namely high spatial resolution satellite data. Statistically robust and transparent approaches for assessing the accuracy of satellite temporal change products have been recommended using approaches that assess pixel level thematic mapping accuracy and the accuracy of areal change estimates (Olofsson *et al*. 2014). These established approaches do not quantify the extraction accuracy of individual objects which is still an area of active research (Möller *et al*. 2013; Persello and Bruzzone 2010; Yan and Roy 2014). Consequently, in this study we assumed that if any field extraction errors did occur then they were systematic for each pair of subsets and so did not unduly affect the change information. Moreover, we ensured that the metrics used to summarise field size change (described below) were non‐parametric and robust to extraction errors.

### Decadal Landsat field size change assessment

The area of each agricultural field object was calculated by counting the number of 30 m pixels it encompassed. The median field size, i.e. the 50th percentile, and also the 25th and 75th percentile field size statistics were computed for each 15 × 15 km subset and for each of the two time periods. Non‐parametric summary statistics, rather than parametric statistics (mean and standard deviation), were used as field size frequency distributions can be skewed (Ferguson *et al*. 1986) and because they are robust to outlying values due to, for example, field extraction errors. The percentage change in the median field size between the earlier and later time periods was computed as [(median_later_ – median_earlier_) / median_earlier_] × 100.

Histograms of the field sizes were computed for each 15 × 15 km Landsat subset and ‘back‐to‐back’ histograms were created for the two time periods using the same optimal histogram binning scheme derived by combining the datasets using the Sturges method (Sturges 1926). The significance of any changes in field size distribution between the two dates was quantified using a bootstrap version of the two‐sample Kolmogorov–Smirnov (KS) test (Conover 1971) that is more robust to the presence of potential ties (Abadie 2002).

In accordance with packing theory (Erdös and Graham 1975), the field sizes are expected to be inversely related to the number of fields if the fields are regularly shaped. To examine this, the field sizes (km^2^) were plotted against the number of fields per cultivated km^2^ for each subset at all locations. To examine if the median field size was related to field size diversity the median field size was compared with the field size interquartile range, defined as the 75th–25th percentile field size.

## Results

### Selected contemporary agricultural field size change locations

Table 1 summarises the seven selected 15 × 15 km subsets. The cereal and biofuel crop subsets were located in continents defined by the FAO 2010 production statistics with the top two greatest crop productions. No Landsat subsets over rice agriculture were selected because even though there were high rice yield locations in all continents (Figure 2) an exhaustive search found no significant unambiguous change in rice field size; this is discussed in more detail below. Landsat subsets were selected within Argentina (soybeans), Brazil (sugarcane), France (wheat), Malaysia (oil palm) and the USA (maize), where yields for the selected crop type exceeded the 80th percentile (Figure 2) and where there was documented change in field size, farm size, agricultural intensity or extent. In addition, subsets in Albania (wheat) and Zimbabwe (wheat) were added as, although they do not exhibit particularly high crop yields or production, they have experienced documented dramatic political and agricultural change. The primary driving force of agricultural field size change is tabulated in Table 1 and the context and likely causes of field size changes are discussed below after the quantitative field size change analysis.

**Table 1:** Summary of selected Landsat subset field size change locations and Landsat acquisition dates

Major crop	Continent (crop rank)	Nation	Locale	15 × 15 km subset centre Lat., Long.	Landsat path/row	Landsat acquisition dates	Primary field size change driver
Soybeans	Americas (1)	Argentina	Argentinian Pampas Córdoba	–32.85°, –62.11°	228/83	02/10/1986 02/15/2011	Technological advancements
Sugarcane	Americas (1)	Brazil	Coastal Brazil northern Paraná	–22.79°, –52.19°	223/76	12/22/1991 05/03/2011	Government policy changes
Wheat	Europe (2)	France	Central France, Poitou‐Charentes	46.78°, –0.01°	200/27	09/28/1984 08/29/1999	Government policy changes
Oil Palm	Asia (1)	Malaysia	Coastal Malaysian peninsular, Perak	4.20°, 101.10°	127/57	12/27/1990 06/01/2010	Government policy changes
Maize	Americas (1)	USA	Corn Belt Plains, Iowa	42.88°, –94.73^o^	28/30	07/27/1989 08/17/2011	Technological advancements
Wheat	Europe	Albania	Northern coastal region	19.45°, 41.97°	186/31	06/10/1991 06/14/2010	Political change
Wheat	Africa	Zimbabwe	Mashonaland East province	–18.47°, 31.60°	169/73	06/22/2001 05/25/2011	Political change

Listed in alphabetical row order by nation, except for the last two locations that were selected without reference to the EarthStat crop yield map. The continental scale global crop rank as defined by 2010 FAO production statistics (tons) for the indicated major crop type in each subset are shown in parentheses.

### Landsat field extractions

**Figure 5:**
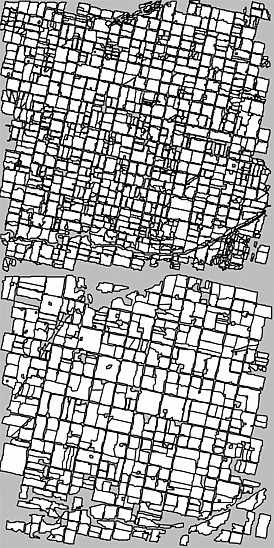
Argentina extracted agricultural field objects (white), for the 15 × 15 km subset area (grey) segmented and classified in eCognition from the two Landsat acquisitions illustrated in Figure 1. Classified field objects composed of less than six 30 m pixels removed as they could not be confidently identified

Figure 5 shows the fields extracted from the Argentinian Pampas subset data, which since the 1990s has become an area of intensive soybean production (Gavier‐Pizarro *et al*. 2011). The two Landsat dates were sensed in mid‐February in the Pampas growing season (USDA 1994) and capture a time period 25 years apart. A 25‐year time period was used in order to capture the agricultural landscape before and after the intensification of soybean production and because the availability of cloud‐free Landsat images acquired in the same growing season was limited for shorter periods.

The locations and extents of the extracted fields (Figure 5) appear correctly identified when compared with the Landsat true colour reflectance data (Figure 1). The extracted fields (white) are surrounded by non‐agricultural areas, including ditches, grassy swards, rivers, roads, and farm buildings. Fields that did not fall completely within the subset were discarded from the subsequent field size analysis. This example illustrates the utility of the object‐based classification to identify agricultural fields and also to provide geographic context for the nature of the field size changes. The field sizes appear to have increased from 1986 to 2011 due to a consolidation of adjacent land parcels and a reduction in the number of fields. The spatial arrangement of the fields has been largely preserved between the two dates. This is likely because of constraints imposed by the historic land use (paved roads and farm buildings are not converted to agricultural fields) and by the landscape structure (the rivers in the North and South of the image).

**Figure 6:**
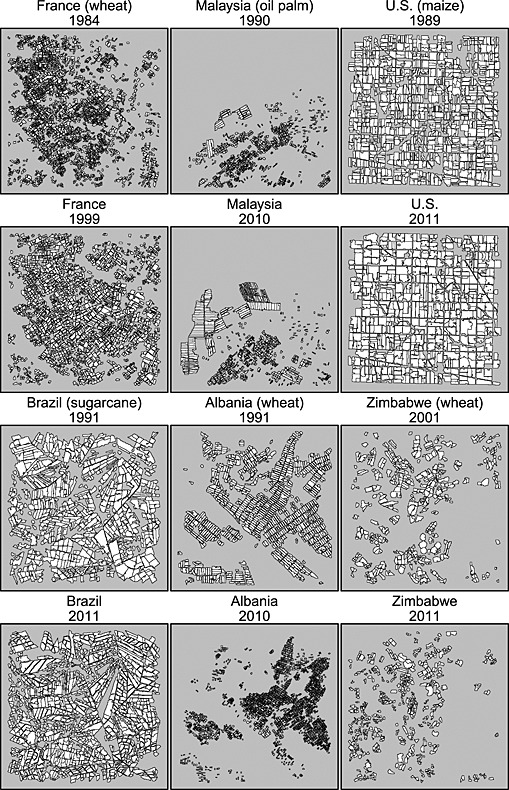
Extracted agricultural field objects (white), for the 15 × 15 km subset areas (grey), for all the study sites (Table 1) except Argentina (already shown in Figure 5). Classified field objects composed of less than six 30 m pixels removed as they could not be confidently identified

**Figure 7:**
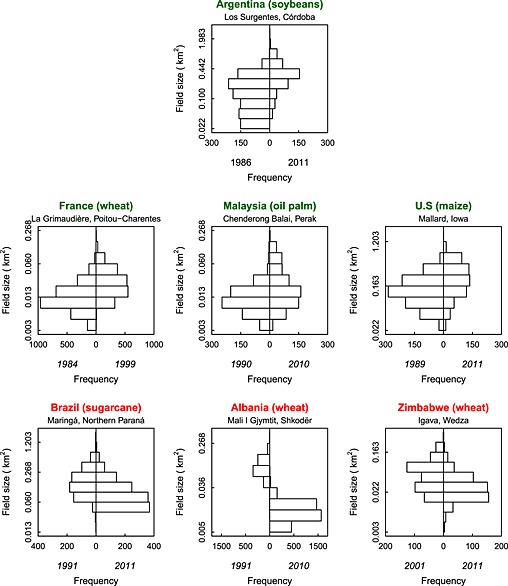
Back to back histograms of field sizes for each 15 × 15 km subset (Table 1). The field sizes (*y* axis) are illustrated with a natural log scale but annotated in km^2^ for visual clarity. The histograms are ordered alphabetically and by field size change – increases in field size (green), and decreases in field size (red)

Figure 6 shows the fields extracted from the two Landsat acquisitions for the other sites (Table 1). The sites in France, Malaysia, and the USA exhibit field size increases whereas the sites in Brazil, Albania, and Zimbabwe exhibit decreases. The smallest fields occurred in the 1984 France, 1990 Malaysia, and in the 2010 Albania Landsat images. A minority of fields were removed from the 1984 France and 1990 Malaysia field segmentations because they had areas less than the six 30 m pixel minimum mapping unit. The greatest number of small fields occurred in the 2010 Albanian Landsat acquisition and we estimate 40% of the cultivated land had fields that could not be unambiguously mapped as they were too small. Some fields were introduced or removed between the dates of the two Landsat acquisitions, this is particularly evident for the Malaysian site, but for all sites the 15 × 15 km subset dimensions were sufficiently large to capture the field size populations in each Landsat acquisition (Figures 5 and 6) and their changes (Figure 7).

### Quantitative decadal field size change analysis

The largest field sizes were in Argentina (2011) and the USA (2011) with 75% percentiles of 0.447 km^2^ and 0.364 km^2^ and maximum field sizes of 2.023 km^2^ and 1.284 km^2^, respectively (Table 2). The magnitudes of these values are similar to those reported by Ferguson *et al*. (1986), who observed maximum field sizes of 0.8 km^2^ (200 acres) for spring wheat fields in Montana. Similarly, Yan and Roy (2014) found a South Dakota median field area to be 0.1053 km^2^ with several fields greater than 3 km^2^. The smallest fields occurred in the later Albanian Landsat subset which was consequently the most difficult to interpret.

**Table 2:** Summary field size change statistics for the seven selected locations (Table I)

Location name	Landsat acquisition years	25th Percentile (km^2^)	50th Percentile (i.e. median km^2^)	75th Percentile (km^2^)	Largest field size (km^2^)	No. of fields	Total field area (km^2^)	No. of fields/ cultivated km^2^	% Change in median field size	Two‐sample KS test	
										D	Crit. D (0.05)
Argentina	1986	0.053	0.123	0.233	0.800	1071	172.055	6.225	159	0.443	0.093
	2011	0.196	0.319	0.447	2.023	436	160.135	2.723			
France	1984	0.014	0.020	0.030	0.235	2697	67.848	39.750	100	0.412	0.048
	1999	0.025	0.040	0.065	0.381	1977	100.348	19.701			
Malaysia	1990	0.013	0.020	0.029	0.400	752	18.868	39.857	45	0.262	0.086
	2010	0.017	0.029	0.058	0.282	676	33.182	20.372			
USA	1989	0.077	0.123	0.203	0.680	964	146.600	6.576	89	0.313	0.085
	2011	0.119	0.233	0.364	1.283	599	160.797	3.725			
Brazil	1991	0.095	0.151	0.242	1.671	674	129.975	5.186	–47	0.357	0.078
	2011	0.056	0.079	0.132	0.884	1202	141.166	8.515			
Albania	1991	0.066	0.087	0.113	0.294	775	73.206	10.505	–86	0.988	0.056
	2010	0.009	0.013	0.015	0.084	2661	35.465	75.031			
Zimbabwe	2001	0.045	0.092	0.132	0.515	438	46.343	9.451	–55	0.379	0.106
	2011	0.031	0.041	0.071	0.495	511	29.267	17.460			

For clarity the field size statistics are shown in km^2^ to the nearest three decimal places and the percentage change in median field size results are expressed to the nearest percent. The minimum field size considered was 0.0054 km^2^. Note that 1 km^2^ = 100 ha. The two‐sample KS tests were significant for all the locations at the 99% confidence level (*p*‐values were < 2.2 × 10^–16^ for all sites).

The results of the bootstrap two sample non‐parametric KS test (Table 2) indicate that the field size distributions changed significantly at all seven locations. This is expected given the location selection criteria and is evident in the back‐to‐back field size histograms (Figure 7). The field size histograms are skewed and so are displayed with a log scale.

**Figure 8:**
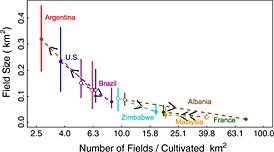
Relationship between field size (*y* axis) and the number of fields per cultivated km^2^ (*x* axis) for all seven Landsat subsets and both acquisition periods. Open and closed circles show the first (earlier) and second (later) Landsat acquisitions with arrows indicating the direction of time progression. The vertical lines show the 25th percentile (bottom) and 75th percentile (top) field size and the circles show the median, i.e. 50th percentile field size. The *x*‐axis labels are graduated on a log10 scale rounded to the nearest decimal place

**Figure 9:**
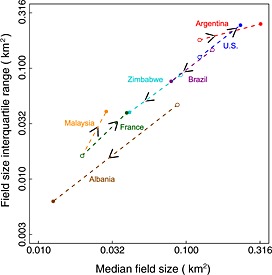
Relationship between interquartile range of field sizes, i.e. 75th–25th percentile (*y* axis) and the median field size (*x* axis) for all seven Landsat subsets and both acquisition periods (labelled and coloured as Fig. 8). The axes labels are graduated on a log10 scale rounded to the nearest two decimal places. Open and closed circles show the first (earlier) and second (later) Landsat acquisitions with arrows indicating the direction of time progression

The field sizes are inversely related to the number of fields (Figure 8). The number of fields per cultivated km^2^ is plotted on the *x* axis with a log scale to capture the considerable variation in the number of fields and cultivated areas among the seven locations. The later Argentinian acquisition (2011) had the smallest number of fields per cultivated km^2^ (2.7) and the second Albania acquisition (2010) had the greatest number (75.0) (Table 2). The circles show the median field sizes and the arrows point from the earlier (first) to the later (second) Landsat subset acquisition date, illustrating where the median fields sizes increased or decreased. The field size interquartile range (75th – 25th percentile) is directly proportional to the median field size (Figure 9). A reduced major axis linear regression fit, used as it allows for both the dependent and independent variables to have error (Cohen *et al*. 2003), provides a relationship of the form interquartile range = 0.104 + (1.123 median field size) with an *R* ^2^ = 0.933. This relationship occurs because field sizes were smaller in the vicinity of roads, buildings, and rivers that segment the landscape. Consequently, subsets where the median field size increase but that retain a number of small fields, due to the landscape structure and pre‐existing land use, have an increased field size interquartile range. Other local factors, including spatial gradients of soil fertility and sub‐surface drainage, and spatial patterns of human tenure and management and farmer decision making, may also play a similar spatial constraining role. However, these factors are not possible to assess from the Landsat data.

Significant *increases* in field sizes occurred in Argentina (soybeans), France (wheat), Malaysia (oil palm), and the USA (maize) (Table 2, Figure 8). The greatest median field size increase was in Argentina where the median increased from 0.123 km^2^ (1986) to 0.319 km^2^ (2011), a 159% increase. In France, the USA, and Malaysia, the median field size increases were 100%, 89%, and 45%, respectively. For all these locations the number of fields per cultivated km^2^ decreased by a factor of about two.

Significant *decreases* in field sizes occurred in Brazil (sugarcane), Albania (wheat), and Zimbabwe (wheat) (Table 2, Figure 8). The greatest field size decrease was in Albania where the median field size decreased from 0.087 km^2^ (1991) to 0.013 km^2^ (2011), an 86% decrease and the number of fields per cultivated km^2^ increased by a factor of about 7. In Brazil and Zimbabwe the median field size decreases were 47% and 55%, respectively, and the number of fields per cultivated km^2^ increased by a factor of about 1.6 and 1.8, respectively (Figure 8, Table 2).

No quantitative decadal field size change analysis was undertaken for rice because, even though there was high rice yield locations in all the continents, an exhaustive search of the global Landsat archive and the available high spatial resolution GoogleEarth time series found no systematic or significant unambiguous rice field changes. The majority of the regions with high rice yields (Figure 2) had fields that were not discernible in Landsat data, primarily because, as illustrated in Figure 3, the fields were too small relative to the Landsat 30 m resolution. Typically, these small fields were rectangular and narrow and only a minority had curvilinear shapes associated with terraced rice cultivation. Detailed examination of 61 globally distributed high‐yield rice cultivation locations discernible in the available high spatial resolution GoogleEarth imagery revealed no changes in rice field area. Certainly, rice field boundaries were sometimes moved but the field areas were not significantly changed. A global minority of very large (more than ~0.2 km^2^) and intermediate sized (typically ~0.05 km^2^) rice fields that were discernible in the Landsat data record were found in regions including California (USA), Arkansas (USA), Sinaloa (Mexico), and New South Wales (Australia), and in regions including Andalucía (Spain), the Po Valley (Italy), and Epirus (Greece), respectively. No large rice field areal change was evident in the post‐1984 Landsat data record. We expect that the high rice yield locations (Figure 2) contain rice that is grown predominantly using irrigated cultivation methods that produce the greatest yields (Maclean *et al*. 2002). Limited studies suggest that the size of irrigated rice fields is constrained by water management issues, and depends on the slope, the soil type, and the water supply flow rate needed to ensure optimal irrigation depth and soil infiltration rates (Anbumozhi *et al*. 1998; Brouwer *et al*. 1988). We hypothesise, therefore, that the lack of any significant observable rice field change is associated with these constraints, i.e. that smaller rice fields evident in the high spatial resolution imagery have already been adapted by farmers to their optimal size and that larger rice fields were established with optimal size.

### Decadal field size change contextual analysis

#### Argentina

The Americas is by continent the largest producer of soybeans and Argentina is the third largest national producer of soybeans within the Americas (after the USA and Brazil) (FAOSTAT 2010). The Landsat data were located in the state of Córdoba, over the northern portion of the Central Pampas, in an area of historical agricultural production that has experienced significant increases in intensive soybean cultivation (Gavier‐Pizarro *et al*. 2011; Viglizzo *et al*. 2001). Genetically modified (GM) soybean varieties were adopted in Argentina from the 1990s to reduce costs and expand production into marginal lands using zero‐till cultivation practices (Craviotti 2002; Pengue 2005; Zak *et al*. 2008). Increased demand for biofuels, and the growth of large agricultural enterprises and one year land leasing encouraged the replacement of pasture and mixed cropping with profit maximising soybean monocultures, creating larger fields that are better suited to mechanised cultivation (Craviotti 2002; Lamers *et al*. 2008; Mathews and Goldsztein 2009; Viglizzo *et al*. 2011). This is unambiguously consistent with the results obtained in this study, where the Argentinean median field sizes exhibited a 159% increase over a 25‐year period from 1986 to 2011.

#### France

Europe is by continent the second largest producer of wheat after Asia, and France is the largest national producer of wheat in Europe (FAOSTAT 2010). High yields of wheat were encouraged after the development of the European Union (EU) (Bouma *et al*. 1998) and were facilitated by high nitrogen fertiliser application rates (Brisson *et al*. 2010). The Landsat subset was located over the province of Poitou‐Charentes in north‐west France in an area of relatively high wheat production. Despite agricultural intensification encouraged by the EU Common Agricultural Policy (CAP) to remove hedgerows and increase field sizes, the fields in France still remain relatively small (Busch 2006; Stoate *et al*. 2009; Thenail and Baudry 2004). Indeed, field size growth is constrained by the prevalence of historic bocage landscape patterns dating to the nineteenth and early twentieth centuries (Thenail and Baudry 2004; van Eetvelde and Antrop 2004). Reforms to the CAP in the 1990s shifted focus away from intensification to an increased emphasis on environmentally friendly agriculture and the adoption of agricultural set‐aside and field margin preservation schemes (Daniel and Perraud 2009; Mosnier *et al*. 2009). As previously noted, the median field size increase for this location was 100% over a 15‐year period from 1984 to 1999.

#### Malaysia

Asia is by continent the largest producer of oil palm and Malaysia is the largest oil palm producing nation globally (FAOSTAT 2010). Oil palm plantations have expanded significantly in the past few decades encouraged by increased global demand for food and biofuel (Abdullah *et al*. 2009; Wicke *et al*. 2011). The Landsat data were located over the eastern end of the Malaysian peninsular, in the state of Perak, an area where existing agricultural lands have been converted to oil palm plantations (Abdullah and Nakagoshi 2006). Since 1985, industrial development driven by government policy reforms, including a 2005 national biofuels policy, has stimulated the domestic conversion of oil palm into biodiesel for export to primarily European markets (Abdullah *et al*. 2009; Abdullah and Nakagoshi 2006; Gan and Li 2008; Wicke *et al*. 2011). An increase in the area of land under palm oil (that exhibits larger field sizes compared with surrounding food crop fields) has been unambiguously driven by changes in government policy, resulting in a 45% median field size increase from 1990 to 2010.

#### USA

The Americas is by continent the largest producer of maize and the USA is the largest national producer (FAOSTAT 2010). Farm sizes (and so potentially field sizes) in the USA have increased in the past few decades due to a shift toward industrialised agriculture (Barlett 1993; Cleveland 1995; Hart 1986; USDA 2009). The Landsat data were located over Iowa in the eastern end of the Corn Belt plains ecoregion that is an area of particularly intensive agriculture, primarily maize and soybeans for animal feed and biofuels (Karr‐Lilienthal *et al*. 2005; Petrou and Pappis 2009). Intensive cash grain production has been facilitated by technological advancements, including selective breeding, genetic manipulation, irrigation, and mechanisation (Plourde *et al*. 2013; USDA and Natural Resources Conservation Service 2007). Similar to farm size increases noted in the literature (MacDonald 2011), the median field sizes increased by 89% over a 22‐year period from 1989 to 2011 and this was driven primarily by technological advancements.

#### Brazil

The Americas is by continent the largest producer of sugarcane and Brazil is the largest national producer (FAOSTAT 2010). Sugarcane, grown primarily for ethanol production, was adopted in Brazil in the 1970s in response to government policies (including the ProAlcool scheme) initiated to reduce fossil fuel dependence (Hira and de Oliveira 2009; Moraes 2011). Although subsidies for ethanol production were withdrawn by 2004 (Uriarte *et al*. 2009) global demand for food, fibre and energy has caused continued production and expansion of Brazilian sugar cane plantations (Smeets and Faaij 2010). The Landsat data were located in Paraná, one of the largest sugarcane producing states, that in recent decades has experienced expansion of sugarcane at the expense of other agricultural and pastoral land uses (Nassar *et al*. 2008). This conversion to sugarcane plantations was observed, with changes in both the field sizes (earlier pasture and agricultural fields were larger than the later sugarcane fields; Figures 6 and 7) and the field arrangement (due to the placement of completely new field boundaries). These changes resulted in an increase in the number of fields and a median field size decrease of about 47% over a 20‐year period from 1991 to 2011.

#### Albania

Albania was considered because of well documented rapid agricultural change. The Landsat data were in the northern coastal agricultural district of Shkodër. Intensive collectivised large‐scale agriculture with guaranteed markets and predominantly wheat crop cultivation was practiced until the collapse of communism in 1991 (Christensen 1994; Cungu and Swinnen 1999). This political change produced a shift from state to private ownership of lands, with land being divided based on household size (Muller and Sikor 2006; Swinnen 1999). Inappropriate tenure agreements led to land fragmentation (Sikor *et al*. 2009; van Dijk 2003) and small field sizes due to limited opportunities for expansion (Muller and Munroe 2008). This is apparent in the Landsat results, with nearly all the large fields subdivided into fields with distinct field boundaries, and a median field size decrease of 86% over a 19‐year period from 1991 to 2010.

#### Zimbabwe

Zimbabwe was considered because of well documented rapid agricultural change. The Landsat data were located over Mashonaland East Province, one of the highest crop production areas in Zimbabwe (Jingura and Matengaifa 2008), with cash (cotton and tobacco) and food crops (primarily irrigated wheat and maize) and also some subsistence farming (Nyagumbo and Rurinda 2012; Palmer 1990; Yates 1980). Until 2000 the greater majority of the commercial agricultural land in Zimbabwe was farmed by colonial, predominantly European, farmers. After independence in 1979, a government‐based process of land reform aimed to redistribute lands more equally on a ‘willing seller, willing buyer’ basis (Palmer 1990). In 2000 the forced seizure and redistribution of land by a fast track process of land reform without compensation was legalised and commercial farmland was redistributed to small‐scale indigenous famers (Cliffe *et al*. 2011). This resulted in the mismanagement of many commercial farms and caused declining yields and cropland degradation (Prince *et al*. 2009; Richardson 2007; Sachikonye 2003). The number of fields per cultivated km^2^ almost doubled due to the introduction of smaller plots constructed within the preserved outer boundaries of pre‐existing field boundaries. A 55% median field size decrease over a 10‐year period from 2001 to 2011 was found for this location, likely driven by the fast track land reforms.

## Discussion

A body of literature has alluded to the likelihood of field sizes changing due to increasing demand for food, fibre and biofuel. Significant changes in field sizes were observed over approximately decadal periods and are likely to have significant ecological and biogeochemical consequences. The magnitude and relative speed of the observed changes were dramatic and greater than changes due to many natural processes.

A pragmatic approach was used to select seven locations where there were cloud‐free Landsat data and that captured contemporary field size change. The reported sample results indicate increasing field sizes associated with technological advancements (improved mechanisation and new and improved crop varieties) and governmental policy changes (economic investment reforms, incentives for agricultural practices, and biofuel mandates). Decreasing field sizes were associated with political events that rapidly changed the agricultural sector and where pastures were converted to arable agriculture. Local patterns of field size change were complex however. Field sizes remained small where there were constraints imposed by the landscape structure and pre‐existing land uses, presumably because these constraints were not easily or profitably removed. Other local factors, including spatial gradients of soil fertility and sub‐surface drainage, and spatial patterns of human tenure and management as well as farmer decisionmaking, may also play a spatially constraining role. However, these factors are not possible to assess from Landsat data and were not possible to ascertain from the available literature. No systematic or significant unambiguous changes in rice field sizes could be detected and this may be due to physical constraints concerning water management issues. This study illustrates that changes may be influenced by a multitude of environmental and human factors, although the attribution of land use change drivers is challenging (Veldkamp and Lambin 2001; Verburg *et al*. 2004).

A pattern of increasing field sizes was indicated by this research. This may decrease landscape spatial complexity and therefore decrease landscape diversity through the homogenisation of land uses. These changes are likely to have significant ecological and biogeochemical consequences. Potential consequences include a reduction in the number of natural and semi‐natural landscape patches (Merriam and Wegner 1992; Petit and Firbank 2006; Pogue and Schnell 2001), declines in biodiversity and loss of habitat (Benton *et al*. 2003; Duro *et al*. 2014; Green *et al*. 2005; Krebs *et al*. 1999), increased Aeolian soil erosion (Skidmore *et al*. 1970), reduced plant–pollinator interactions (Gabriel and Tscharntke 2007), modification of the ability of invasive species to establish themselves (Holway 2005; Yates *et al*. 2004), increased likelihood of disease pathogens and pests (Margosian *et al*. 2009), and loss or degradation in buffers to nutrient, herbicide and pesticide flows from agricultural lands (Martin 2011; Ryszkowski 1992). Evidence also suggests that larger regular‐shaped fields are more likely to be irrigated and therefore have increased water consumption (O'Brien *et al*. 1998; Schuck and Green 2001). Field size increases have been associated with agricultural intensification (Kuemmerle *et al*. 2013; Tscharntke *et al*. 2005) that is associated with increased agricultural water and energy use and emission of greenhouse gases (Foley *et al*. 2005; Matson *et al*. 1997; Robertson *et al*. 2000).

While this research provides a snapshot of field size changes, the extrapolation of results to infer reliable, more general patterns will depend on the number of sample locations and their placement relative to the heterogeneity of field size distributions and changes. As this information is not well defined, global wall‐to‐wall quantitative spatially explicit field size mapping is suggested. Terrestrial changes may be exhibited and understood in quite different ways in the context of multi‐decadal rather than decadal time series, and so repeated decadal field size mapping is suggested. It is expected that more Landsat data acquired in the 1970s to the present day will become available as they are repatriated from non‐US receiving stations into the US Landsat archive. A limitation of the current research is the 0.0054 km^2^ minimum mapping unit that results from the 30 m Landsat pixel size and that precludes small field identification. In regions that are, or have previously been dominated by small holder agriculture, such as in Asia (Fan and Chan‐Kang 2005) and Africa (Morton 2007), this may be an issue when Landsat data are used. As higher spatial resolution satellite alternatives to Landsat are not available prior to 1999, a potential solution is to use historical aerial photography such as declassified military imagery (Tappan *et al*. 2000).

The absolute accuracy of the resulting field extractions was unknown but, given the interactive and visual extraction approach used, the field extractions reflect the highest accuracy we judged possible. We admit that if other operators followed the same approach the resulting field maps could be different, particularly for smaller fields. The minimum field size that can be extracted reliably from satellite data is dependent on several factors, including the sensor spatial resolution, satellite geolocation errors, the spectral contrast between field interiors and exteriors, the field shape, and the extraction methodology. The use of a minimum field size threshold is problematic for locations where field sizes changes occur above and below the threshold. In this research both the consolidation of small adjacent fields into larger fields, and the subdivision of larger fields into smaller ones was observed. However, the percentage change in the median field size will only be sensitive to small field size detection issues if 50% or more of the field sizes were undetectable. Of the seven sites considered, only the second Landsat image acquired over the Albanian site had more than a minority of fields (and less than 50%) that were close to the 0.0054 km^2^ minimum field size limit and so the changes in Albanian field sizes from 1991 to 2010 were sufficient to reveal an unambiguous decrease.

Future agricultural production is expected in many regions to rely on increased agricultural yield rather than agricultural land expansion (Erb *et al*. 2013), although yield increases are likely to vary geographically and with crop type, and to be sensitive to climate changes (Lobell and Field 2007). This research suggests that in such regions future field sizes may also increase, likely facilitated by opportunities provided by technological developments, and driven by the need to increase agricultural yield and by demand for particular crops in response to macroeconomic drivers and governmental policies. However, the extent to which field sizes globally have already increased is unquantified.
